# Patient treatment and outcome after breast cancer orbital and periorbital metastases: a comprehensive case series including analysis of lobular versus ductal tumor histology

**DOI:** 10.1186/s13058-020-01309-3

**Published:** 2020-06-26

**Authors:** Martin Blohmer, Li Zhu, Jennifer M. Atkinson, Sushil Beriwal, Joshua L. Rodríguez-López, Margaret Rosenzweig, Adam M. Brufsky, George Tseng, Peter C. Lucas, Adrian V. Lee, Steffi Oesterreich, Rachel C. Jankowitz

**Affiliations:** 1 Department of Pharmacology & Chemical Biology, University of Pittsburgh, Pittsburgh, PA USA; 2 Charité – Universitätsmedizin Berlin, Berlin, Germany; 3 Women’s Cancer Research Center, UPMC Hillman Cancer Center, Magee Women’s Research Institute, Pittsburgh, PA USA; 4 Department of Biostatistics, University of Pittsburgh, Pittsburgh, PA USA; 5 University of Pittsburgh School of Medicine, Department of Radiation Oncology, UPMC Hillman Cancer Center, Pittsburgh, PA USA; 6 School of Nursing, University of Pittsburgh, Pittsburgh, PA USA; 7 University of Pittsburgh School of Medicine, Department of Medicine, Division of Hematology/Oncology, UPMC Hillman Cancer Center, Pittsburgh, PA USA; 8 Department of Pathology, University of Pittsburgh, Pittsburgh, PA USA; 9 Department of Medicine, Division of Hematology/Oncology, Perelman School of Medicine, Abramson Cancer Center, University of Pennsylvania, Philadelphia, PA USA; 10 Rena Rowan Breast Center, Perelman Center for Advanced Medicine and the Abramson Cancer Center, 3rd Floor, West Pavilion, 3400 Civic Center Boulevard, Philadelphia, PA 19104 USA

**Keywords:** Breast cancer, Invasive lobular carcinoma, Metastasis, Eye, Ophthalmology

## Abstract

**Background:**

Breast cancer is the most common malignancy to spread to the orbit and periorbit, and the invasive lobular carcinoma (ILC) histologic subtype of breast cancer has been reported to form these ophthalmic metastases (OM) more frequently than invasive ductal carcinomas (IDC). We herein report our single academic institution experience with breast cancer OM with respect to anatomical presentation, histology (lobular vs. ductal), treatment, and survival.

**Methods:**

We employed the natural language processing platform, TIES (Text Information Extraction System), to search 2.3 million de-identified patient pathology and radiology records at our institution in order to identify patients with OM secondary to breast cancer. We then compared the resultant cohort, the “OM cohort,” to two other representative metastatic breast cancer patient (MBC) databases from our institution. Histological analysis of selected patients was performed.

**Results:**

Our TIES search and manual refinement ultimately identified 28 patients who were diagnosed with breast cancer between 1995 and 2016 that subsequently developed OM. Median age at diagnosis was 54 (range 28–77) years of age. ER, PR, and HER2 status from the 28 patients with OM did not differ from other patients with MBC from our institution. The relative proportion of patients with ILC was significantly higher in the OM cohort (32.1%) than in other MBC patients in our institution (11.3%, *p* = 0.007). Median time to first OM in the OM cohort was 46.7 months, and OM were the second most frequent first metastases after bony metastases. After diagnosis of the first distant metastasis of any kind, median survival of patients with ILC (21.4 months) was significantly shorter than that of patients with IDC (55.3 months, *p* = 0.03). Nine patients developed bilateral OM. We observed a significant co-occurrence of OM and central nervous system metastases (*p* = 0.0053). The histological analysis revealed an interesting case in which the primary tumor was of a mixed ILC/IDC subtype, while only ILC was present in the OM.

**Conclusions:**

OM from breast cancer are illustrative of the difference in metastatic behavior of ILC versus IDC and should be considered when treating patients with ILC, especially in those with complaints of visual acuity changes.

## Background

With over 1.5 million new cases each year, breast cancer accounts for 25% of all cancer cases in women worldwide [1]. Breast cancer is a heterogenous disease, both molecularly and histologically. The invasive lobular carcinoma (ILC) histologic subtype represents 10–15% of all breast cancer, constituting the second most common subtype after invasive ductal carcinoma (IDC) [2].

ILC is marked by its characteristic loss of the cell adhesion molecule E-cadherin. In classical ILC, invasive cells grow in single file pattern with little disruption of the stroma [3]. ILC is also commonly characterized by pathological features that are usually associated with a good prognosis, such as hormone receptor (HR) positivity, human epidermal growth factor receptor (HER2/neu) negativity, low proliferative rate, and low histological grade [4], yet it has been described to have a higher propensity for late distant metastases compared to IDC tumors of similar grade and hormone receptor status [5, 6].

The relevance of differentiating outcome in patients with ILC versus IDC histological subtypes stems from their reported unique clinical presentations, including their sites of metastases, survival rates, and responses to systemic therapy. While few studies directly compare therapeutic response in patients with ILC vs. IDC, response to neoadjuvant chemotherapy has been reported to be lower in patients with ILC compared to patients with IDC [7], and the proportional advantage of adjuvant aromatase inhibitors over tamoxifen is reported to be higher for patients with ILC compared to patients with IDC [8]. Moreover, patients with ILC have a 50% higher risk of death after 10 years in comparison to patients with IDC [9]. Finally, we and others have shown unique patterns of metastases for patients with ILC, with ILC more likely to metastasize to the ovaries and gastrointestinal tract, and IDC more likely to metastasize to the lung and the liver [10–12].

Ophthalmic metastases (OM) are rare and often caused by carcinomas like breast cancer, lung cancer, and hepatocellular carcinoma [13, 14]. Breast cancer is reported to be responsible for the most OM, with it being the primary neoplasms of origin of 40% of the metastases to the orbit and periorbit [13]. These metastases can occur in all compartments in and around the eye. Intraocular metastases are found most frequently in the choroid, which is likely due to its high degree of vascularity [15]. Periocular metastases have also been observed and have a distinct clinical presentation [16].

In a recent literature review, Tsagkaraki et al. described 40 cases in which ILC caused OM [17]. This propensity of ILC to metastasize to the ophthalmic region is underlined by other studies that investigated OM caused by any histological subtype and reported higher rates of ILC that led to OM [18–20], but these studies are limited by small sample size. We employed a natural language processing platform called TIES (Text Information Extraction System) [21] recently described by Jacobsen et al. [22], to search all pathology and radiology reports in a large institutional database. With access to over 2.3 million patients within our UPMC health system with archived radiology or pathology reports [22], we set our objective to test whether OM were more frequent in ILC than in IDC. Further emphasis was put on anatomical features, treatment, and outcome of these cases according to histology.

## Methods

### Text Information Extraction System (TIES)

Due to the rarity of OM, we took a unique approach in identifying affected patients. The Text Information Extraction System (TIES) developed by investigators within the Department of Biomedical Informatics at the University of Pittsburgh provided us with the ability to search for specific terms or phrases in pathological and radiological reports of all patients treated at UPMC hospitals. All reports in TIES are de-identified. As a natural language processing platform, TIES translates simple search terms into ontologies which are used to search through patient reports. These ontologies consist of the NCI Metathesaurus’ synonyms and abbreviations, thus making the search for a specific term not limited by word arrangement or spelling. Temporal combination of two queries consisting of many different ontologies gave us the ability to search for patients that were diagnosed with breast cancer in one report and with OM in another.

### Clinical data

This retrospective case series does not represent a comprehensive review of all patients seen at our institution, as the initial search through TIES was limited to cases with available pathology and radiology reports. Eligibility criteria were (A) breast cancer confirmed in a pathology report and (B) metastatic involvement of structures in and around the eye suspected clinically and confirmed in either a radiology or pathology report. A filter was set to only include female patients to reduce false positive results. The search criteria in TIES were purposefully broad as to not exclude any OM caused by breast cancer. The search results in TIES were then manually validated to ensure that criteria A and B were met.

After obtaining institutional review board approval from the University of Pittsburgh (IRB Number PRO15050502), we requested identified data for patients in whom the TIES search indicated primary breast cancer and OM. Clinical notes, pathology reports, radiology reports, therapeutic regimens, and outcome for these patients were manually reviewed by members of the investigative clinical team. We will hereafter refer to the resultant group of patients identified by the TIES search and validated by manual chart review as the “OM cohort.”

Patient and tumor characteristics for the OM cohort, including histological subtype, estrogen receptor (ER), progesterone receptor (PR), HER2/neu receptor status, involvement of axillary lymph nodes, and initial stage at diagnosis were obtained from available clinical and/or pathology reports. Outcome information including identification of metastatic sites was obtained via review of the treating oncologist’s notes, radiology reports, and/or pathology reports. Sites of metastases were grouped into eye, lung, bone, liver, central nervous system (CNS), distant lymph nodes, ovary, peritoneum, gastrointestinal tract, skin, local recurrence, and “other” metastatic sites.

We next compared primary breast tumor characteristics and outcome from the OM cohort to two other large representative cancer patient databases from our institution. First, we used the UPMC Network Cancer Registry, a prospectively curated institutional database of all cancer patients seen at UPMC, to collect information on patients diagnosed with breast cancer between January 1, 1990, and June 1, 2018, who subsequently developed distant metastases. We also compared the OM cohort to a second institutional breast cancer database, the Metastatic Breast Cancer Database*.* This database includes patients diagnosed with breast cancer and distant metastases and has been prospectively curated at UPMC Magee-Women’s Hospital between January 1, 1999, and November 31, 2018.

### Immunohistochemistry

Tumor tissue from three patients identified through TIES was available for histological analysis. Formalin Fixed, Paraffin Embedded (FFPE) paired primary tumor and OM tissue was available from one patient; only OM tissue was available for the other two patients. Tissue sections were cut (4 μm) and stained, one with hematoxylin and eosin (H&E), one with an E-cadherin antibody, and one with an estrogen receptor (ER) antibody. For antibody staining, the slides were deparaffinized, rehydrated, and stained using a standard histology protocol. Antigen retrieval was performed using a citrate buffer (Dako, Carpinteria, CA) in a decloaking chamber at 123 °C before being stained using an Autostainer Plus (Dako) platform with TBST rinse buffer (Dako). The E-cadherin antibody (Mouse monoclonal – 4A2C7, Invitrogen, Carlsbad, CA) was applied using a 1:500 dilution at room temperature followed by a secondary antibody of Mach 2 Mouse HRP (Biocare Medical, Pacheco, CA). The ER antibody (Mouse monoclonal – 1D5, Dako) was applied using a 1:100 dilution at room temperature followed by a secondary antibody anti-mouse HiDef HRP Polymer System (Cell Marque, Rocklin, CA). Pictures were taken using a × 200 magnification with the software SPOT imaging.

### Statistical analysis

Time to first OM was calculated as the time between initial diagnosis of breast cancer and the first diagnosis of metastatic involvement of the orbital or periorbital structures. Disease-free survival (DFS) was calculated as the time from initial breast cancer diagnosis until the first recurrence, while distant metastasis-free survival (DMFS) was defined as the time between the initial breast cancer diagnosis and first diagnosis of a distant metastasis. Survival after OM was calculated as the time between first diagnosis of an OM and death or last follow-up for censored patients. Overall survival (OS) was calculated as the time between diagnosis of the primary breast cancer and death or last follow-up for censored patients.

*p* values for continuous variables were calculated using the Wilcoxon rank sum test; Fisher’s exact test was used for categorical variables, and the log-rank test for survival. Unknown data was removed in all tests. Survival probabilities were estimated using the Kaplan-Meier method. R (3.5.1) was used for all statistical analysis.

## Results

### Identification of cases through TIES

**Fig. 1:**
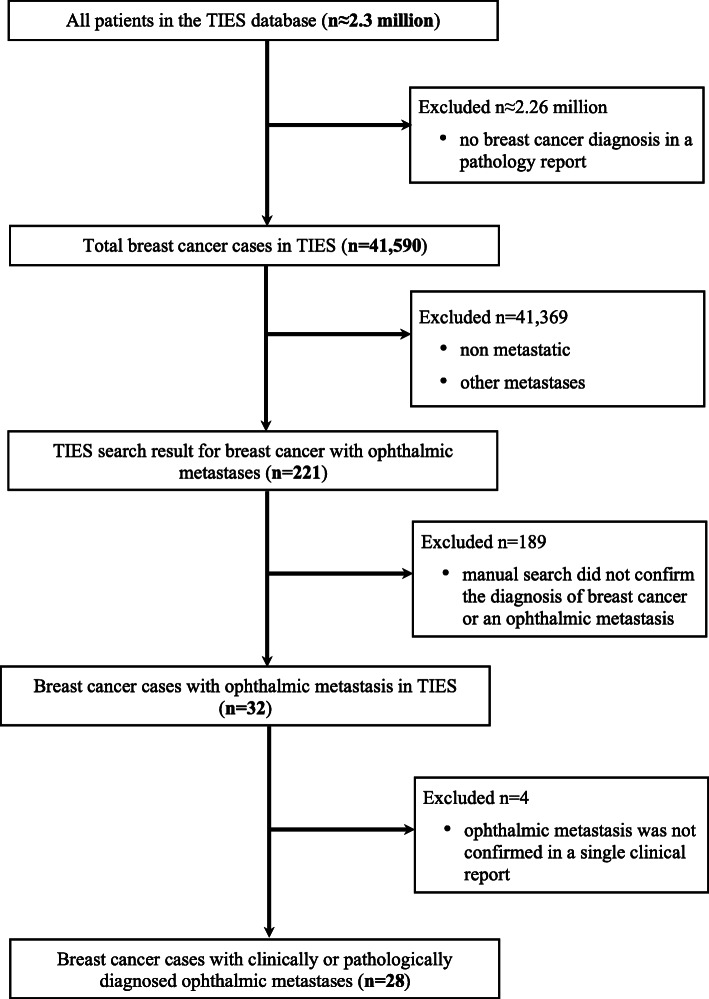
Consort diagram of patient selection

The TIES search yielded 41,590 female breast cancer patients diagnosed between 1981 and 2018. Search criteria of breast cancer and OM yielded 221 cases, but the initial search resulted in a large amount of false positive results. A manual review of the TIES data for these cases eliminated 189 cases in which either primary breast cancer or OM could not be confirmed. Identified data from the remaining 32 patients was then analyzed via a more comprehensive review of the patient medical records. OM originating from a breast primary could not be confirmed in four cases, and those cases were thus excluded. The final resultant OM patient cohort with confirmed breast cancer metastatic to the orbit or periorbit included 28 cases diagnosed between 1995 and 2016 (Fig. 1).

### Patient characteristics

Patient characteristics of the OM cohort are shown in Table 1. The median age at initial diagnosis of breast cancer was 55 (range 28–77 years). ER and PR status were positive in 75.0% and 60.7% of the primary tumors, respectively, and HER2/neu status was positive in 10.7% of cases. The histological subtype of the primary tumor was IDC in 14/28 of the patients (50%), ILC in 9/28 of the patients (32.1%), and mixed ILC/IDC subtype in 2/28 tumors (7.1%). The histological subtype of 3/28 tumors (10.7%) could not be identified.

**Table 1:** Patient characteristics and clinicopathological features of tumors

	Breast cancer primaries with ophthalmic metastasis identified through TIES (absolute number + percentage) *N* = 28	Metastatic breast cancer in the UPMC Network Cancer Registry (absolute number + percentage) *N* = 1366	*p* value
**Age**			
Median age at diagnosis of primary	55	53	0.4455
< 50 at diagnosis	10 (35.7%)	520 (38.1%)	0.847
≥ 50 at diagnosis	18 (64.3%)	846 (61.9%)	
**Race**			1
White	26 (92.9%)	1248 (91.4%)	
Black	2 (7.1%)	104 (7.6%)	
Others	0	14 (1.0%)	
**Survival (median in month)**			
Overall survival	82.1	58.8	0.47
Disease-free survival	26.4	35.4	0.52
Distant metastasis-free survival	34.2	36.6	0.76
**Histological subtypes**			0.00067
IDC	14 (50.0%)	1167 (85.4%)	
ILC	9 (32.1%)	155 (11.3%)	
Mixed	2 (7.1%)	44 (3.2%)	
Unknown	3 (10.7%)	0	
**Stage**			0.00106
Stage 0		11 (0.8%)	
Stage I	0 (0%)	340 (24.9%)	
Stage II	5 (17.9%)	573 (41.9%)	
Stage III	7 (25.0%)	186 (13.6%)	
Stage IV	7 (25.0%)	184 (13.5%)	
Stage unknown	9 (32.1%)	72 (5.3%)	
**Lymph node status**			0.04927
Positive	18 (64.3%)	814 (59.6%)	
Negative	2 (7.1%)	386 (28.3%)	
Unknown	8 (28.6%)	166 (12.2%)	
**ER status**			0.2597
ER+	21 (75.0%)	981 (71.8%)	
ER−	4 (14.3%)	385 (28.2%)	
Unknown	3 (10.7%)	0	
**PR status**			0.2809
PR+	17 (60.7%)	805 (58.9%)	
PR−	6 (21.4%)	519 (38.0%)	
PR unknown	5 (17.9%)	42 (3.1%)	
**HER2 status**			1
HER2/neu +	3 (10.7%)	51 (3.7%)	
HER2/neu −	20 (71.4%)	277 (20.3%)	
Equivocal	0	9 (0.7%)	
HER2/neu unknown	5 (17.9%)	1029 (75.3%)	

*IDC* invasive ductal carcinoma, *ILC* invasive lobular carcinoma, *ER* estrogen receptor, *PR* progesterone receptor, *HER2/neu* human epidermal growth factor receptor

Comparison of the patient and primary tumor characteristics from the OM cohort to those patients with any metastases from breast cancer in the UPMC Network Cancer Registry can be found in Table 1. The relative proportion of patients with ILC and/or mixed histology was significantly higher in the OM cohort compared to all patients with MBC in the UPMC Network Cancer Registry (*p* = 0.0007). No statistically significant differences in ER, PR, and HER2/neu receptor status between these two groups were found. Patients with OM were more likely to have higher stages at diagnosis (*p* = 0.0011) and higher frequency of axillary lymph node involvement (*p* = 0.0492) compared to other patients with MBC in the UPMC Network Cancer Registry.

### Anatomical presentation

**Fig. 2:**
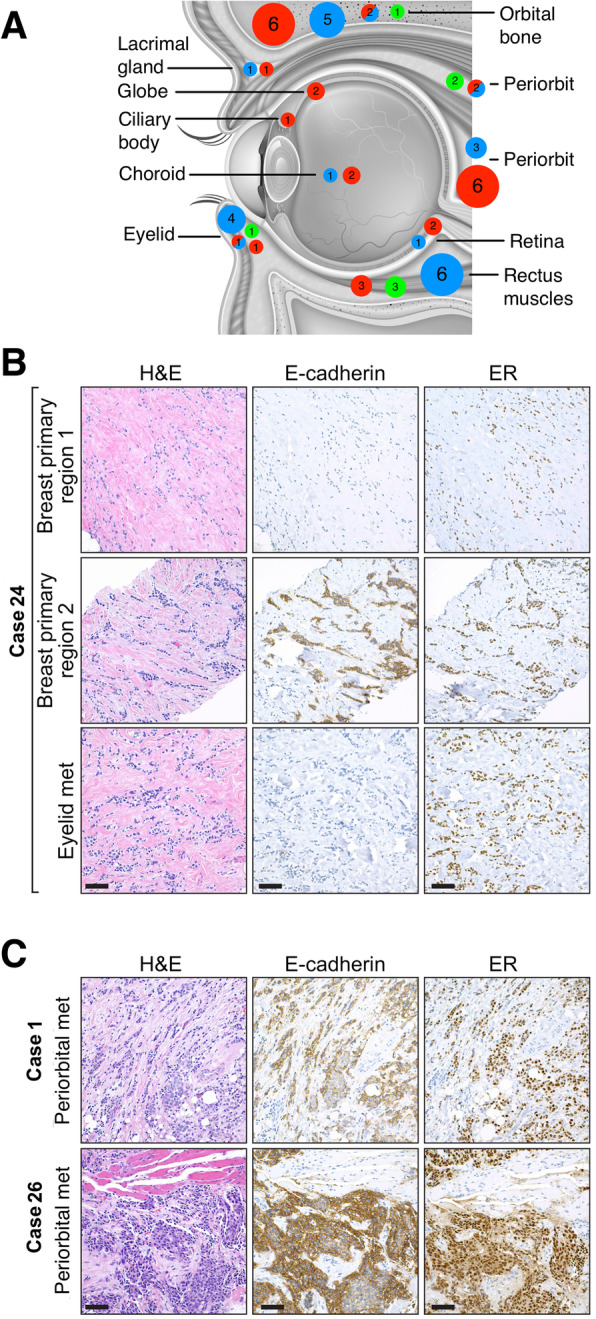
Anatomy and histology of OM. **a** Representation of the anatomical location of all OM. Red dots represent metastases from an IDC primary, blue dots represent metastases from an ILC primary, mixed red and blue dots represent metastases from a mixed IDC/ILC primary, and green dots represent metastases from a primary of unknown histological subtype. Numbers indicate how many patients were affected by OM to this location. In cases where patients had OM to multiple locations within the ophthalmic region, each location was displayed separately. Graphic courtesy of Shutterstock [23]. **b** Histologic analysis of the primary breast tumor for case 24 shows a mixed pattern of invasive lobular and ductal carcinoma. The majority of the tumor is composed of lobular carcinoma, showing single infiltrating cells and linear cords of cells dissecting stroma, which are negative for E-cadherin staining but positive for ER (top row). A discrete, although minor component of the tumor is composed of somewhat larger infiltrating cells forming clusters; these clusters are strongly E-cadherin positive and ER positive (middle row). The biopsy of metastatic disease in the left lower eyelid shows only lobular pattern metastatic carcinoma that is E-cadherin negative and ER positive (bottom row). **c** Two other cases of periorbital metastatic breast cancer in unrelated patients are shown. For these cases, the primary breast cancer specimens were not available for review. In both cases, the histologic features of metastatic carcinoma show some lobular pattern infiltration, particularly for case 1, but features that are more in keeping with metastatic ductal carcinoma. E-cadherin staining is strongly and diffusely positive for both cases, and both are ER positive. Scale bar = 100 μm

In the OM cohort, 10/28 patients (35.7%) had metastases limited to the left eye, and 9/28 patients (32.1%) had a metastasis limited to the right eye. Interestingly, 9/28 patients (32.1%) had metastatic involvement of both eyes. Of these patients, 4/9 had ILC, 1/9 had IDC, 2/9 had mixed ILC/IDC tumor subtype, and 2/9 had unknown tumor subtype. Most (78.6%) of patients in the OM cohort had OM not effecting the globe, 14.3% had metastases that were exclusively intraocular, and 7.1% had tumors with metastatic involvement of ophthalmic tissue inside and outside of the globe. Of the metastases outside of the globe, the majority (11/22, 50%) were in the rectus muscles. Four of these metastases to the extraocular muscles originated from a primary that was solely comprised of IDC. Of the 6 patients with intraocular metastases, 3 had metastases to the optic nerve and 3 had metastases to the choroid. Metastases to the eyelid were reported in 5/28 patients (17.9%). A detailed representation of the location of all OM is displayed in Fig. 2a.

### Histological analysis

We performed a histological analysis of three cases from the OM cohort (OM 1, 24, and 26) who had available tumor tissue. For case 24, two samples of the primary breast tumor and one biopsy of the OM was available for review (Fig. 2b). The two breast primary tumor specimens were primarily composed of ILC but also contained IDC cells. We thus confirmed the initial diagnosis of a mixed ILC/IDC subtype. However, interestingly, only ILC cells were present in the paired OM for this patient, as was evident by their negative staining for E-cadherin and their single file growth pattern (Fig. 2b). Biopsies of the OM for cases 1 and 26 were also available for analysis **(**Fig. 2c). Case 1’s primary tumor was of IDC origin according to pathology reports, and the OM also displayed IDC histology. The histological subtype of the primary tumor of case 26 is not known; the OM stained strongly for E-cadherin, suggesting IDC histology.

### Survival analysis

**Fig. 3:**
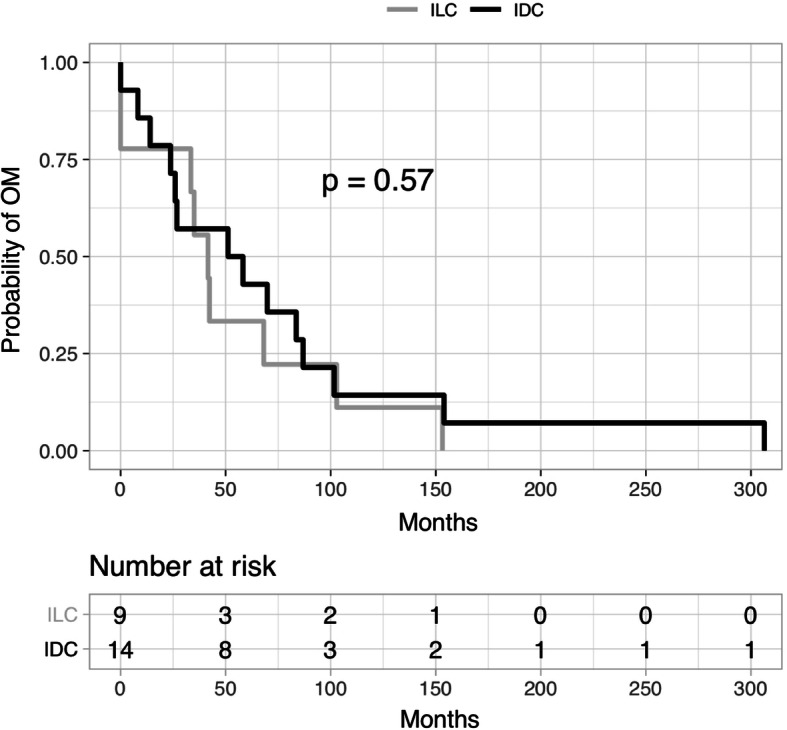
Probability of first ophthalmic metastasis in patients with IDC and ILC

**Fig. 4:**
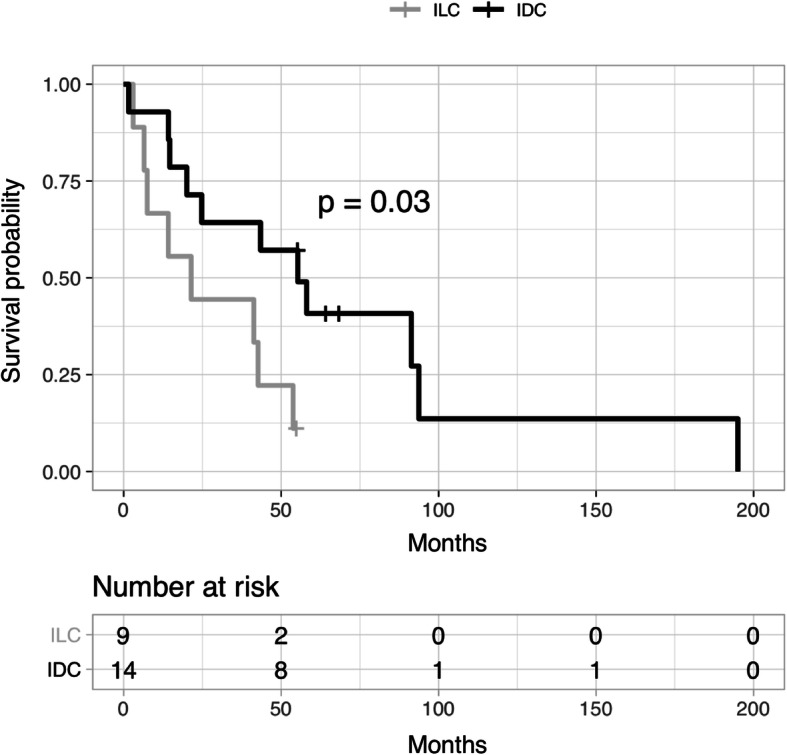
Survival after first metastasis in patients with IDC and ILC

At a median follow-up of 78.4 months after diagnosis of primary breast cancer, 2/28 patients from the OM cohort were still alive (7.1%), 24/28 were confirmed to be deceased (85.7%), and 2/28 patients were lost to follow-up (7.1%). Median overall survival was 82.1 months, with a median DFS of 26.4 months and a median DMFS of 34.2 months. There is no significant difference in OS, DFS, and DMFS in the OM cohort compared to other patients with MBC in the UPMC Network Cancer Registry (Table 1). Median time to first OM was 46.7 months, which was not statistically different between patients with ILC vs. IDC (Fig. 3). Time to first OM in the OM cohort was not significantly different than time to other sites of metastasis in the Metastatic Breast Cancer Database (Additional file 1). DMFS was longer (35.05 months) for patients with ILC compared to patients with IDC (23.34 months) in the OM cohort, supporting a tendency for late relapse for ILC. However, after a diagnosis of a first distant metastasis of any kind, the remaining median survival of patients with ILC (21.4 months) was significantly shorter than that of patients with IDC in the OM cohort (55.3 months, *p* = 0.03) (Fig. 4).

### Metastatic pattern

**Fig. 5:**
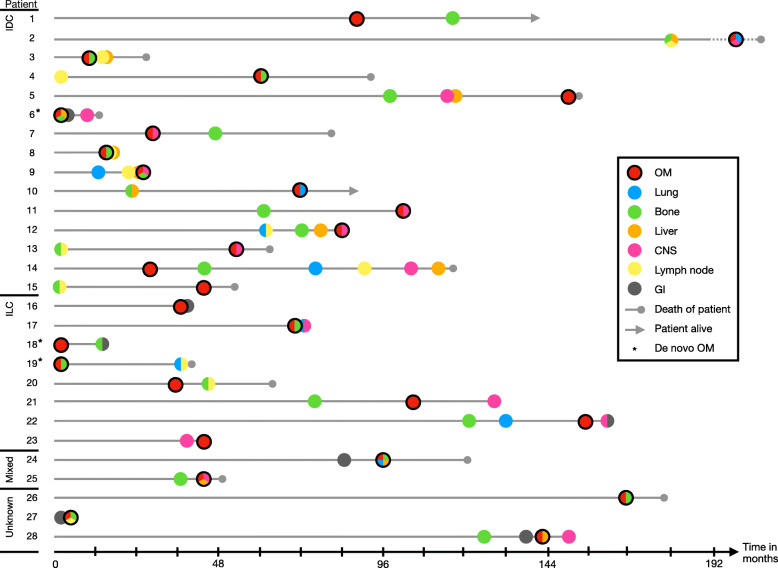
Metastatic sequence for patients in the OM cohort. For each patient, the time from diagnosis of primary breast cancer to the diagnosis of a new metastatic site is displayed. Metastatic sites are color coded as follows: OM: red, lung: blue, bone: green, liver: orange, CNS: pink, distant lymph node: yellow, gastrointestinal tract: gray. If multiple metastatic sites were diagnosed within 1 month, they are displayed with a circle in multiple, corresponding colors. Solid gray circle at the end of timeline indicates death of patient; timelines for patients that were alive at the time of analysis end with an arrow. An asterisk after the patient number denotes the presence of de novo OM

In the OM cohort, 12/28 patients (42.9%) developed OM as their first distant metastasis (Fig. 5). Out of the seven patients with de novo stage IV disease, three had OM at the initial presentation. OM were the second most frequent site of first distant metastasis after bone, which was the first site of distant metastasis in 15/28 patients (53.6%). Involvement of distant lymph nodes was the first site of distant metastasis in 5/28 patients (17.9%). While only 2/28 (7.1%) patients had CNS involvement when they were diagnosed with distant metastatic disease, 13 additional patients later developed CNS metastases as a second or subsequent metastatic event. This proportion of patients 15/28 (53.6%) in the OM cohort that developed CNS metastases throughout the course of their disease is significantly higher than the 420/1495 (28.1%) of patients in the Metastatic Breast Cancer Database that developed CNS metastases (*p* = 0.0053). CNS metastases preceded the diagnosis of OM in 2/15 patients (13.3%), they were diagnosed after the OM in 6/15 patients (40.0%), and finally they were diagnosed concurrently with OM in 7/15 patients (46.7%). Of note, only 4 of the 15 CNS metastases (26.7%) originated from an ILC primary. The most frequent sites of distant metastases throughout the course of the disease other than the ophthalmic region were bone (16/28; 92.9%), CNS (15/28; 53.6%), liver (12/28; 42.9%), distant lymph nodes (12/28; 42.9%), and lung (9/28; 32.1%). A detailed representation of the chronological sequence of metastatic progression for all 28 patients is displayed in Fig. 5 (detailed data in Additional file 2). The earliest metastases after diagnosis of the primary breast cancer in the OM cohort were bony metastases (median 43.6 months), followed by OM (median 46.7 months), and then metastases to distant lymph nodes (median 55.0 months). These were then succeeded by CNS metastases (median 63.0 months), liver metastases (median 69.4 months), and lung metastases (median 70.4 months).

### Clinical management

Of the 28 patients with OM, 14 received adjuvant hormonal therapy. The number of lines of systemic therapy for metastatic disease for all patients in the OM cohort can be seen in Table 2. Anastrozole was the most frequently administered first-line therapy for advanced disease (to 7 patients), followed by Fulvestrant (to 4 patients) and then Tamoxifen, Capecitabine, Letrozole, Docetaxel, and Paclitaxel (the latter 5 were all administered to 3 patients as first-line therapy). Second-line therapies were most frequently Fulvestrant, Capecitabine, and Anastrozole (to 5, 4, and 3 patients respectively) while as third-line therapies, Fulvestrant was administered most frequently (to 6 patients), followed by Tamoxifen, Docetaxel, and Mitoxantrone (all administered to 3 patients). ER-positive and ER-negative patients were included in this analysis.

**Table 2:** Description of individual patients in OM cohort

Patient	Histology of primary breast cancer	Site of first distant metastasis	Time to first distant metastasis in months	Location of OM	Time to OM in months	Side of OM	Location of XRT therapy to OM	Survival in months after OM	CNS metastasis	Number of systemic therapy lines for metastatic cancer
1	IDC	Ophthalmic	86.8	Orbital bone, Rectus muscles	86.8	Right	Orbital bone	55.8 (alive)	No	2
2	IDC	Adrenal glands, bone, CNS, distant lymph nodes, liver	178.1	Choroid, ciliary body	294.1	Right	Choroid	79.0	Yes	8
3	IDC	Ophthalmic, bone	8.2	Retro-orbital fat, globe, orbital bone	8.2	Right	Orbital bone	20.1	No	5
4	IDC	Distant lymph node	14.3	Rectus muscles, lacrimal gland, orbital bone	58.2	Right	Orbital bone	35.5	No	11
5	IDC	Bone, spleen	96.0	Retro-orbital fat	153.8	Bilateral	No XRT	0.2	Yes	6
6	IDC	Ophthalmic, bone, liver	0	Orbital bone	0	Left	No XRT	14.3	Yes	3
7	IDC	Ophthalmic, CNS	26.8	Choroid, retina	26.8	Right	Choroid	55.2	Yes	3
8	IDC	Ophthalmic, bone	14.1	Orbital bone	14.1	Left	Orbital bone	1.7	No	0
9	IDC	Lung	10.9	Retina	23.8	Left	Retina	1.8	Yes	3
10	IDC	Bone, liver	20.7	Orbital bone, Periorbital soft tissue	69.8	Left	Orbital bone	22.8 (alive)	No	5
11	IDC	Bone	59.1	Orbital soft tissue	101.6	Left	No XRT	1.0	Yes	4
12	IDC	Distant lymph nodes, lung	59.8	Globe	83.5	Left	No XRT	1.1	Yes	8
13	IDC	Contralateral breast, distant lymph nodes	0	Eyelid, periorbit	51.1	Left	Eyelid	Lost to follow-up	Yes	4
14	IDC	Ophthalmic	26.0	Rectus muscle	26.0	Right	No XRT	91.3	Yes	6
15	ILC	Bone, distant lymph nodes	0	Orbital bone, eye lid, lacrimal gland	41.6	Bilateral	Orbital bone, eyelid	12.1	No	8
16	ILC	Ophthalmic	35.0	Rectus muscles	35.0	Left	No XRT	3.0	No	1
17	ILC	Ophthalmic, bone	70	Rectus muscle	70.0	Right	Orbital bone	4.6	Yes	2
18	ILC	Ophthalmic, peritoneum	0	Retina, choroid, rectus muscle	0	Left	No XRT	14.1	No	0
19	ILC	Ophthalmic, bone	0	Rectus muscles	0	Bilateral	No XRT	41.3	No	5
20	ILC	Ophthalmic	33.4	Eyelid, periorbital soft tissue, orbital bone	33.4	Bilateral	Both eyelids	21.5	No	2
21	ILC	Bone	74.0	Rectus muscle, orbital bone	102.8	Bilateral	Orbital bone	Lost to follow-up	No	5
22	ILC	Bone, contralateral breast, thyroid gland	119.2	Orbital bone, eyelid	153.1	Right	Orbital bone	8.7	Yes	7
23	ILC	CNS	36.6	Retrobulbar soft tissue	42.2	Left	Twice retrobulbar	1.8	Yes	3
24	Mixed IDC and ILC	Rectum	82.6	Rectus muscles, eyelid	94.0	Bilateral	Retrobulbar, eyelid	24.7	No	5
25	Mixed IDC and ILC	Bone	34.9	Orbital bone	41.6	Bilateral	Orbital bone	8.7	Yes	3
26	Unknown	Ophthalmic, bone	165.0	Orbital bone, rectus muscles	165	Right	Orbital bone	14.2	No	0
27	Unknown	Gastrointestinal tract, peritoneum	0	Rectus muscles, eyelid, orbital bone	2.8	Bilateral	Retrobulbar	4.3	No	2
28	Unknown	Bone	122.7	Orbital and periorbital soft tissue	140.5	Bilateral	Retrobulbar	10.0	Yes	3

*XRT* radiation therapy

We identified 20/28 patients in the OM cohort (71.4%) with available radiation records. Four of these patients received radiation therapy more than once, sometimes to different sites (see Table 2), resulting in 25 separate treatments. The most common locations treated were the entire orbit (13/25, 52%), followed by the eyelid (5/25, 20%), retrobulbar area (4/25, 16%), and intraocular area (3/25, 12%). The median dose prescribed was 30 Gray (Gy) (range 10–60 Gy) in 12 (range 2–30 fractions) fractions. The most common radiation technique utilized was 3D-conformal for 16/25 treatments (64%), electron for 5/25 treatments (20%), intensity-modulated radiation therapy (IMRT) for 3/25 treatments (12%), and 1 patient received stereotactic body radiation therapy (SBRT) (4%).

## Discussion

Our initial TIES search of a database including over 2.5 million patient records led to the identification of 28 patients with OM from breast cancer, making our study the largest to date of these events. The use of the natural language processing platform TIES enabled high sensitivity to identify these extremely rare breast cancer metastases.

OM can present in many ways and most often indicate poor prognosis [24, 25]; 92% of our patients in the OM cohort died from metastatic breast cancer. Of all tumors types, breast cancer is responsible for most OM [13], and the ILC histological subtype has been described to cause OM more commonly than IDC [18, 20]. We set out to test this hypothesis in a large institutional cohort and to further characterize patient experience in the setting of OM via a descriptive analysis of their histologic, anatomic, treatment and survival data.

Compared to 1366 patients in the UPMC Cancer Network registry, we observed higher rates of lymph node positivity and higher stages at diagnosis in the OM cohort. We also observed a significant association of OM with a diagnosis of an ILC primary. This confirms earlier reports and emphasizes the need to take OM into consideration when treating patients with ILC, especially in those with complaints of visual disturbances.

A study conducted by Parrozzani et al. reported that patients with choroidal metastases are more likely to have an ER-positive and PR-positive primary breast cancer than patients with breast cancer metastasizing to any other location [26]. In a recent cohort description of orbital metastases by Sindoni et al., they were all ER positive [27]. In our study, ER, PR, and HER2/neu receptor status of primary tumors that led to OM did not differ significantly from the receptor status of other primary breast tumors in a large institutional database of patients with metastatic breast cancer.

OM were diagnosed as the first site of distant metastasis in 12 of 28 patients in the OM cohort, making them the second most frequent site of first distant metastasis after bony metastases. In their literature review, Tsagkaraki et al. have described changes in vision, swelling, and pain as the most frequent symptoms of OM [17], and physicians should consider OM in patients with these complaints. In a comparison with site specific data from the Metastatic Breast Cancer Database, the time between diagnosis of primary breast cancer and OM (median 46.7 months) did not differ significantly from the time to development of distant metastasis to any other site. The sequence of appearance of new metastatic sites in the OM cohort was bony metastases, OM, distant lymph nodes, CNS, liver, and lung; however, such analysis is hampered by limited sample size. Survival after diagnosis of the first distant metastasis was significantly shorter for patients with ILC than for patients with IDC in the OM cohort. In an earlier study focusing on metastatic breast cancer by histology, we found no such difference in outcome after diagnosis of distant metastasis between ILC and IDC [10]. The overall survival in the OM cohort (median 82.1 months), however, did not differ from the survival of other patients with metastatic breast cancer in the two other databases at our institution. Similar overall survival has also been reported in the literature [28]. This leads us to the conclusion that OM are not a sign of shortened OS from time of diagnosis when compared to breast cancer that metastasizes to other locations. However, early detection and treatment of OM should be a priority for treating physicians, as impaired vision leads to a deterioration of quality of life [29]. Additionally, survival times were short in many of the patients after a diagnosis of OM.

Many of the OM in the cohort were in extraocular tissue, with half of these infiltrating the extraocular muscles. In 4 of these cases, the metastasis originated from an IDC breast primary tumor. We are aware of only 5 such cases previously reported in the English literature [30] and thus believe that the TIES search for OM at our institution allowed for an extremely high degree of sensitivity for identification of these rare cases.

Bone was the most frequent other site of distant metastasis in the 28 patients with OM, followed by metastases to the central nervous system (53.6%). While brain metastases have been identified as a risk factor for choroidal metastases [31], our study is, to our knowledge, the first to report an association between metastases to orbit and periorbit and metastases to the CNS. We observed that CNS metastases were most often diagnosed at the same time or after the diagnosis of OM in our cohort. This observation supports consideration of increased screening for CNS metastases in patients with OM.

While small sample size for this study limits our ability to draw strong conclusions, several findings from this work are worthy of clinical consideration and future investigations. The co-occurrence of OM and CNS metastases was less pronounced in ILC in our cohort; only 4 of the 15 CNS metastases were caused by an ILC primary. This may indicate a different pattern of spread for OM from ILC versus from IDC. We speculate that ILC cells are biologically distinct and thus metastasize to the ophthalmic region via different mechanisms than IDC cells, but larger studies are needed to address this question.

Of the 28 OM, 9 were bilateral. Of these 9, 4 originated from ILC and 2 from a mixed IDC/ILC subtype, showing a trend for ILC being more likely to metastasize to both eyes. Interestingly, in one of the patients with a mixed IDC/ILC primary breast tumor, the OM was comprised solely of ILC cells. This adds further support to unique mechanisms of metastasis to the orbit and periorbit for ILC. Raape et al. have speculated that a high amount of estrogen produced in the eye provides a favorable niche for breast cancer metastases, and more specifically ILC [17]. This hypothesis is supported by reports of high ER expression in the retina, lens ciliary body, and iris [32] and high aromatase expression in the lens epithelial cells [33]. However, 15% of the OM in our cohort arose from ER-negative primary tumors, demonstrating the need for further investigation of this hypothesis through analysis of additional clinical samples, and mechanistic analyses.

Given the retrospective case series analysis of our study, we were dependent on available clinical, radiology, and pathology notes. Not all OM in our cohort were histologically confirmed; in many cases, we only had histological information from the primary tumor. However, our study has some notable strengths. We have been able to identify a large number of patients with a rare condition via the natural language processing platform TIES. We were also able to analyze clinical notes and modes of therapy in addition to radiological and histological reports, thus providing a broad understanding of patients with OM as unique metastases from breast cancer.

## Conclusions

We provide a comprehensive description of OM originating from breast cancer, including data on histology, anatomical presentation, treatment, and survival. OS of patients in the OM cohort was similar to that of patients with other metastases. Our experience supports an increased association of OM from breast cancer with the ILC histological subtype, and survival after diagnosis of a first distant metastasis was significantly shorter for patients with ILC vs. IDC in our OM cohort. Furthermore, we show an association between OM and CNS metastases and a considerable number of cases with bilateral OM, points that are important to consider when treating patients with OM.

## Supplementary information

**Additional file 1.** Comparison of time from diagnosis of the primary tumor to a site-specific first metastases. Displayed is a comparison of the time to first ophthalmic metastasis with the time to first metastasis at a specific anatomical site. Data on the time to first metastasis at a specific anatomical site was abstracted from the Metastatic Breast Cancer Database.

**Additional file 2.** Time to Metastasis by Site for Individual Patients.

## Data Availability

The datasets generated during and/or analyzed during the current study are available from the corresponding author on reasonable request.

## Abbreviations

OM: Ophthalmic metastasis
ILC: Invasive lobular carcinoma
IDC: Invasive ductal carcinoma
CNS: Central nervous system
HER2/neu: Human epidermal growth factor receptor 2
ER: Estrogen receptor
PR: Progesterone receptor
DFS: Disease-free survival
DMFS: Distant metastasis-free survival
OS: Overall survival
Gy: Gray
