# Supplementary material for: Patient treatment and outcome after breast cancer orbital and periorbital metastases: a comprehensive case series including analysis of lobular versus ductal tumor histology
Source: Breast Cancer Res. 2020 Jun 26;22:70. doi: 10.1186/s13058-020-01309-3 (PMC7318761; doi:10.1186/s13058-020-01309-3)
Supplement: Supplementary file 1 — Additional file 1. Comparison of time from diagnosis of the primary tumor to a site-specific first metastases. Displayed is a comparison of the time to first ophthalmic metastasis with the time to first metastasis at a specific anatomical site. Data on the time to first metastasis at a specific anatomical site was abstracted from the Metastatic Breast Cancer Database. [file 13058_2020_1309_MOESM1_ESM.pdf]

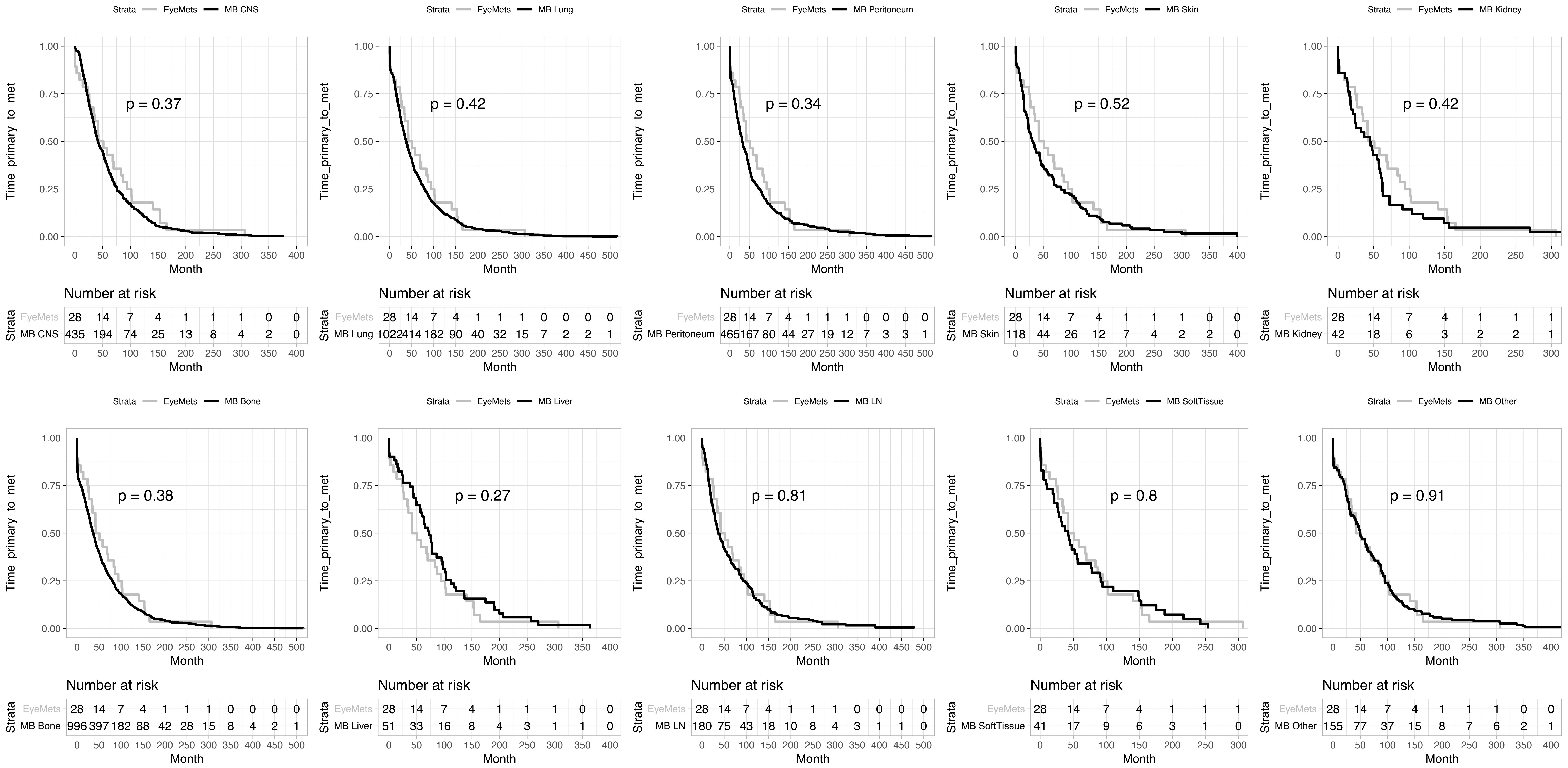
Strata EyeMets MB CNS
p = 0.37
Time_primary_to_met
Month
Number at risk
EyeMets 28 14 7 4 1 1 1 0 0
MB CNS 435 194 74 25 13 8 4 2 0
Strata EyeMets MB Lung
p = 0.42
Number at risk
EyeMets 28 14 7 4 1 1 1 0 0 0 0
MB Lung 1022414 182 90 40 32 15 7 2 2 1
Strata EyeMets MB Peritoneum
p = 0.34
Number at risk
EyeMets 28 14 7 4 1 1 1 0 0 0 0
MB Peritoneum 465167 80 44 27 19 12 7 3 3 1
Strata EyeMets MB Skin
p = 0.52
Number at risk
EyeMets 28 14 7 4 1 1 1 0 0
MB Skin 118 44 26 12 7 4 2 2 0
Strata EyeMets MB Kidney
p = 0.42
Number at risk
EyeMets 28 14 7 4 1 1 1
MB Kidney 42 18 6 3 2 2 1
Strata EyeMets MB Bone
p = 0.38
Number at risk
EyeMets 28 14 7 4 1 1 1 0 0 0 0
MB Bone 996 397 182 88 42 28 15 8 4 2 1
Strata EyeMets MB Liver
p = 0.27
Number at risk
EyeMets 28 14 7 4 1 1 1 0 0
MB Liver 51 33 16 8 4 3 1 1 0
Strata EyeMets MB LN
p = 0.81
Number at risk
EyeMets 28 14 7 4 1 1 1 0 0 0 0
MB LN 180 75 43 18 10 8 4 3 1 1 0
Strata EyeMets MB SoftTissue
p = 0.8
Number at risk
EyeMets 28 14 7 4 1 1 1
MB SoftTissue 41 17 9 6 3 1 0
Strata EyeMets MB Other
p = 0.91
Number at risk
EyeMets 28 14 7 4 1 1 1 0 0
MB Other 155 77 37 15 8 7 6 2 1
