# Supplementary material for: Patient treatment and outcome after breast cancer orbital and periorbital metastases: a comprehensive case series including analysis of lobular versus ductal tumor histology
Source: Breast Cancer Res. 2020 Jun 26;22:70. doi: 10.1186/s13058-020-01309-3 (PMC7318761; doi:10.1186/s13058-020-01309-3)
Supplement: Supplementary file 2 — Additional file 2. Time to Metastasis by Site for Individual Patients. [file 13058_2020_1309_MOESM2_ESM.docx]

| **Time (Months) to Metastasis by Site for Individual Patients** | | | | | | | | | | |
| --- | --- | --- | --- | --- | --- | --- | --- | --- | --- | --- |
| **Patient** | **Histology** | **OM** | **Lung** | **Bone** | **Liver** | **CNS** | **LN** | **GI** | **OS** | |
| 1 | IDC | 86.9 | - | 114.6 | - | - | - | - | 142.0 |  |
| 2 | IDC | 306.3 | 273.2 | 178.2 | 178.2 | 306.3 | 178.2 | - | 373.2 |  |
| 3 | IDC | 8.3 | - | 8.3 | 13.2 | - | 12.0 | - | 28.3 |  |
| 4 | IDC | 58.3 | - | 58.3 | - | - | 0 | - | 93.7 |  |
| 5 | IDC | 153.9 | - | 96.1 | 115.0 | 113.0 | - | - | 154.2 |  |
| 6 | IDC | 0 | - | 0 | 0 | 7.6 | - | 1.9 | 14.2 |  |
| 7 | IDC | 26.8 | - | 45 | - | 26.8 | - | - | 82.1 |  |
| 8 | IDC | 14.1 | - | 14.1 | 15.3 | - | 14.6 | - | 15.6 |  |
| 9 | IDC | 23.8 | 10.9 | 24.1 | 22.7 | 23.8 | 19.7 | - | 25.6 |  |
| 10 | IDC | 69.8 | 69.8 | 20.7 | 20.7 | - | - | - | 89.1 |  |
| 11 | IDC | 101.6 | - | 59.2 | - | 101.6 | - | - | 102.6 |  |
| 12 | IDC | 83.6 | 59.8 | 70.3 | 75.8 | 83.6 | 59.8 | - | 84.6 |  |
| 13 | IDC | 51.2 | - | 0 | - | 51.2 | 0 | - | 64.1 |  |
| 14 | IDC | 26 | 74.3 | 41.8 | 110.2 | 102 | 88.6 | - | 117.7 |  |
| 15 | ILC | 41.7 | - | 0 | - | - | 0 | - | 53.8 |  |
| 16 | ILC | 35.0 | - | - | - | - | - | 37.5 | 38.0 |  |
| 17 | ILC | 68.2 | 71 | 68.2 | - | 71 | - | - | 74.6 |  |
| 18 | ILC | 0 | - | 12.5 | - | - | - | 13 | 14.2 |  |
| 19 | ILC | 0 | 35 | 0 | - | - | 35 | - | 41.3 |  |
| 20 | ILC | 33.5 | - | 43 | - | - | 43 | - | 64.9 |  |
| 21 | ILC | 102.9 | - | 74.1 | - | 126.8 | - | - | 128.9 |  |
| 22 | ILC | 153.2 | 129.9 | 119.2 | - | 160.7 | - | 161.7 | 161.9 |  |
| 23 | ILC | 42.3 | - | - | - | 36.7 | - | - | 44.1 |  |
| 24 | Mixed | 94.1 | 94.1 | 94.1 | 94.1 | - | - | 82.7 | 121.8 |  |
| 25 | Mixed | 41.6 | - | 34.9 | 42.1 | 41.6 | - | - | 50.2 |  |
| 26 | Unknown | 165.1 | - | 165.1 | - | - | - | - | 179.3 |  |
| 27 | Unknown | 2.8 | - | 2.8 | - | - | 2.8 | 0 | 7.1 |  |
| 28 | Unknown | 140.6 | - | 122.8 | 140.6 | 150.0 | - | 135.8 | 150.6 |  |
